# Mold-Free Manufacturing of Ultra-Thin Composite Film with Flower-like Microstructures for Highly Sensitive Tactile Sensing

**DOI:** 10.3390/ma18122863

**Published:** 2025-06-17

**Authors:** Xin-Hua Zhao, Ling-Feng Liu, Qinyu He, Qi-Jun Sun

**Affiliations:** 1 School of Intelligent Manufacturing and Electrical Engineering, Guangzhou Institute of Science and Technology, Guangzhou 510540, China; 2 School of Physics and Optoelectronic Engineering, Guangdong University of Technology, Guangzhou 510006, China

**Keywords:** composite film, tactile sensors, wearable electronics, health monitoring, electronic skin

## Abstract

Wearable tactile sensors with high sensitivity can be potentially used to continuously monitoring physiological signals that are closely related to disease diagnosis and health condition tracking. However, the development of such tactile sensors involves a number of challenges, including a series of expensive patterning processes for microstructure manufacturing and addressing the large thickness of the microstructured composite film. Herein, a mold-free approach is presented to develop an ultra-thin ZnO/PEDOT:PSS composite film with flower-like microstructures via a feasible solution process for highly sensitive tactile sensors. The fabricated tactile sensors exhibit a high sensitivity of 4 × 10^3^ kPa^−1^ in the pressure range 0–10 kPa, a fast response to various pressures in merits of the hierarchical microstructures on top of the ultra-thin composite films. Thanks to the fascinating performance of the devices, the tactile sensors are demonstrated with the ability to monitor physiological signals, subtle human body motions, and spatial pressure distribution.

## 1. Introduction

In past decades, flexible tactile sensors have attracted tremendous attention because of their potential applications in real-time health monitoring, robotics, human–machine interactions, and electronic skin (e-skin) [1,2,3,4]. According to the types of sensing mechanisms, flexible tactile sensors can be divided into piezoresistive, capacitive, piezoelectric, and triboelectric types, and piezoresistive tactile sensors have been investigated broadly due to their simplified device construction and promising potential for commercialization [4,5]. For future applications, flexible tactile sensors should have high sensitivity, good flexibility, excellent durability, and high resolution for spatial pressure distribution as well. However, the limitations of existing flexible tactile sensors hinder their applications as e-skin for soft robots and wearable health monitoring. Firstly, the majority of the reported pressure sensors have limited flexibility because of the physical properties of the pressure active films or the substrate, restricting sensors from being placed on heavily curved surfaces, such as the joints of a robotic hand or a conical fingertip [6]. Secondly, most of the reported pressure sensor arrays for spatial pressure distribution detection have unsatisfied detection resolution because the sensing pixel usually has a size of hundreds of millimeters, which is much larger than the fingerprint of a human being (~200 µm). Therefore, it is of critical significance to develop ultra-flexible tactile sensors and tactile sensor arrays for their promising potential applications.

For diverse applications, the high sensitivity of the tactile sensors is strictly required, and developing microstructures on pressure-sensitive films has been demonstrated as an effective approach to realize the aforementioned high sensitivity because the microstructures can increase the change of the contact area effectively [7,8]. Currently, the micropatterned structures can be realized by employing the silicon mold, sandpaper template, or natural leaf based molds [9,10,11,12,13]. Traditionally, the conductive polymer composite inks (conductive filler mixed with the polymer matrix) have been coated on the substrate, and after a thermal annealing process, the microstructured pressure-sensitive films can be obtained after the removal of the molds. For the template-assisted fabrication of microstructured composite film, the wastage of composite inks cannot be ignored. Recently, some mold-free approaches for microstructured composite films have been proposed, such as 3D printing [14]. The thickness and softness of both substrate and pressure-sensitive films are important in the development of conformable tactile sensors. However, the coated sensing polymer composites by templates or 3D printing usually have high viscosity, and the resulting micropattered pressures-sensitive films are normally very thick [14,15,16,17,18]. The large thickness of the pressure-sensitive layers hinders their application for conformable electronics, and their pressure detection accuracy is usually affected by bending forces.

For e-skin applications, flexible tactile sensors should be flexible enough and the density of the pressure-sensing pixels should be high enough, which can enable the pressure sensor arrays to reveal the spatial pressure distribution in high resolution. Although in the previously reported works tremendously flexible pressure sensor arrays have been demonstrated, most of the reported pressure sensor pixels have an area size of 1 cm^2^, which is much larger than the size of a human fingerprint. Here, a mold-free construction strategy is proposed to develop pressure sensors for conformable pressure detection and high-resolution spatial pressure distribution mapping. In previously reported works, the polymer composite films for pressure sensors are usually realized by mixing the conductive inorganic materials into the flexible polymer matrix. Therefore, the obtained polymer composite can maintain the good flexibility of the polymer and the excellent conductivity of the inorganic fillers. However, it is difficult to produce ultra-thin pressure sensitive films with microstructures for flexible tactile sensors [19,20,21]. In this work, microstructured pressure-sensing films are fabricated by incorporating the conductive polymer (PEDOT:PSS) and inorganic materials as the skeleton. In brief, the flower-structured zinc oxide (ZnO) on ultra-thin indium tin oxide (ITO)-coated polyethylene terephthalate (PET) substrate is fabricated firstly. After that, a thin layer of PEDOT:PSS film is spray-coated on top of the ZnO film as the conductive layer. Thus, an ultra-thin ZnO/PEDOT:PSS film with microstructures is obtained. Furthermore, the microstructured ZnO/PEDOT:PSS film and the ITO electrodes can be patterned by employing a laser cutting technique for pressure sensor array construction. With ultra-thin thickness (several micrometers) and soft mechanical properties, the fabricated pressure sensor and pressure sensor array could be easily curved on the conformable surface of diverse objects for diverse applications. Additionally, their potential applications in monitoring biological signals in daily life and continuously recording wrist pulse signals are successfully demonstrated. Moreover, the pressure sensor array is employed for spatial pressure distribution mapping, indicating its promising potential as artificial skin for future soft robots.

## 2. Materials and Methods

Materials: The zinc acetate (Zn(CH_3_COO)_2_) was bought from Sigma Aldrich (St. Louis, MO, USA). The PEDOT:PSS conductive film was bought from Xi’an Polymer Light Technology Corp. (Xi’an, China). Indium-tin oxide (ITO)-coated poly(ethylene terephthalate) (PET) sheets (75 µm) were bought from Huanan Xiangcheng Co., Ltd. (Shenzhen, China). The sodium hydroxide (NaOH) and cetyltrimethylammonium bromide (CTAB) were supplied by Sigma-Aldrich.

Preparation and characterization of ZnO and composite film: The synthesis was performed by a hydrothermal reaction of Zn(CH_3_COO)_2_ and NaOH (0.8 M) in the presence of CTAB at 90 °C for 3 h. The concentration of Zn(CH_3_COO)_2_ in deionized water (DI water) was 0.2 M. The PEDOT:PSS polymer was spray-coated from a height of 100 mm, using a pass speed of 50 mm s^−1^ onto the ZnO to form the ZnO/PEDOT:PSS composite film after thermal annealing at 100 °C for 30 min. The crystallinity of the flower-like ZnO film was characterized by X-ray diffractometer (XRD, Bruker, D8 Phaser, Billerica, MA, USA) using Cu Kα radiation (λ = 1.5418 Å) with a scan rate of 2°/min. The surface morphology of ZnO and ZnO/PEDOT:PSS composite film was measured by scanning electron microscopy (SEM, 15 kV, JEOL, Tokyo, Japan, and JSM-6490).

**Figure 1:**
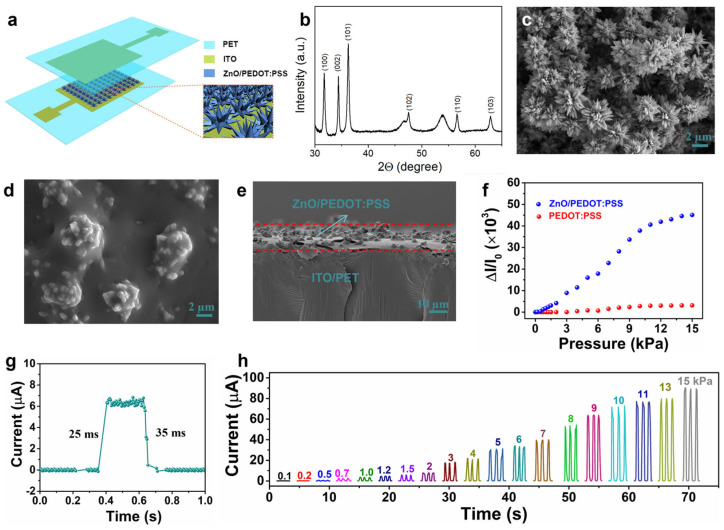
(**a**) Schematic device structure of the pressure sensor. (**b**) XRD of ZnO. (**c**) SEM image of flower-like ZnO materials on ITO/PET substrate. (**d**) SEM image of microstructured ZnO/PEDOT:PSS composite film. (**e**) Cross-sectional view of ZnO/PEDOT:PSS on ITO/PET substrate. (**f**) Relative current responses to diverse pressures of the tactile sensors with ZnO/PEDOT:PSS composite film or pure PEDOT:PSS film. (**g**) Response and relaxation time under a pressure of 1.5 kPa. (**h**) Dynamic pressure responses of the tactile sensors.

Fabrication and characterization of the tactile sensors: For the tactile sensors, ZnO/PEDOT:PSS-coated ITO/PET substrate was cut into proper sizes, and two pieces of the composite films were assembled face to face by 3M tape (T8030c-0). After that, copper wires were attached to the ITO electrodes for external measurement. The schematic sensor structure is shown in Figure 1a. For the tactile sensor arrays, the ZnO/PEDOT:PSS composite films were patterned by ultraviolet laser (355 nm) etching with an etching rate of 100 cm/s at a power of 3 W. Herein, in merits of the excellent sensing performance of our tactile sensor, 6 × 6 tactile sensor arrays were fabricated for spatial pressure mapping. In detail, the ITO electrode patterns on the PET substrate were obtained by laser etching, where the sensing area was 5 × 5 mm^2^. After that, the ZnO microstructures and PEDOT:PSS layer were deposited, subsequently, followed by the construction of the sensor array using a conventional crossbar configuration. Thin copper wires were connected to each ITO bar for external connections. The tactile sensor arrays were formed by laminating the composite films face to face using 3M tapes. The current responses of the tactile sensor and tactile sensor arrays to external pressures were characterized by a pressure sensor characterization system, including a Keithley 2400 source meter (OR, USA) and Mark-10 force test stand (FL, USA). The voltage applied to the tactile sensor and tactile sensor arrays was 1 V.

## 3. Results

### 3.1. Fabrication of Tactile Sensors

In this work, our unique strategy is to fabricate the ultra-thin and microstructured pressure-sensitive films excluding any lithographical patterning or film transfer processes. In short, two manufacturing approaches are included in the fabrication process of the mold-free manufacturing of ultra-thin tactile sensing films. Firstly, the inorganic skeleton (flower-like ZnO) of the tactile sensing film is achieved by a hydrothermal method. The XRD of flower-like ZnO can be found in Figure 1b, showing the classical ZnO crystalline (JCPDS:36-1451). After that, the PEDOT:PSS polymer conductive layer is spray-coated on top of the flower-like ZnO. The realized flower-like ZnO/PEDOT:PSS conductive composite film can maintain the microstructures of the ZnO skeleton and the excellent flexibility of the polymer.

The flower-like ZnO can grow on top of the ITO electrodes without a mold, which is different from the traditional approach to achieve the microstructures of the pressure-sensitive layer for highly sensitive tactile sensors. The size and the height of the ZnO flower are several micrometers, which indicates the potential of the strategy raised in this study to achieve the ultra-thin, microstructured ZnO for high-performance tactile sensors. A conformal coating of PEDOT:PSS on top of the flower-like ZnO by spray printing and no additional film transfer process is required. The surface morphology of the flower-like ZnO is characterized via SEM, as shown in Figure 1c, indicating an effective approach to obtain the microstructured skeleton by using the hydrothermal strategy. The surface morphology of the pressure-sensitive films is important to enhance the sensitivity of the flexible tactile sensors. Here, the morphology of ZnO/PEDOT:PSS composite film is also characterized as shown in Figure 1d. It indicates that the microstructures of the ZnO skeleton is well maintained after coating a thin layer of PEDOT:PSS. The cross-sectional SEM image of the ZnO/PEDOT:PSS composite film is shown in Figure 1e. Additionally, the thickness and conductivity of the spray-printed PEDOT:PSS film could be adjusted by changing the spray-printing times. The electrical conductivity of the ZnO/PEDOT:PSS composite film increases with the increased thickness of the PEDOT:PSS, and the height of the surface microstructures of the composite films decreases with the increased thickness of the PEDOT:PSS film, respectively. In this work, the PEDOT:PSS film with a coating time of 3 s was selected for flexible tactile sensors. The quantitative relationship between the film thickness and the sensing performance of the tactile sensor will be carried out in our future work.

### 3.2. Sensing Performance of the Ultra-Thin Tactile Sensors

The sensing mechanism is the pressure-dependent resistance change in the tactile sensor, thereby a changed current under a constant applied voltage. Distinguished with the tactile sensors based on the flat composite films, the conductive pathways and the contact area both can be changed by the external pressure, which means the resistance is more easily changed. In brief, there are few contacts between the top and bottom tactile sensing layers, resulting in a large degree of resistance in the device. When there is a tiny degree of pressure applied to the tactile sensor, the contact area of these two layers will be slightly increased. If the external pressure is increased further, there will be more conducting pathways formed between these two layers and the resistance will be greatly decreased. The total resistance (R_T_) of the tactile sensor includes the resistance of the tactile sensing films (R_F_) and the contact resistance (R_C_) between these two film layers. The R_T_ of the tactile sensors is very large when there is no external pressure applied to the tactile sensor because of the few contacts between the active layers. However, when an external pressure is applied onto the flexible tactile sensor, the conducting pathways and the contact area increase quickly, resulting in a greatly decreased R_C_. Therefore, the tactile sensor is capable of detecting the external pressure by measuring the changes in current. Compared with the previously reported results, the resistance of the tactile sensors in this work is more easily changed by the applied pressure as shown in Figure 1f,h. It should be noted that the thickness of the tactile sensing layers is much smaller than the previously reported pressure sensors based on the conductive composite, indicating its promising potential for conformal e-skin applications.

**Figure 2:**
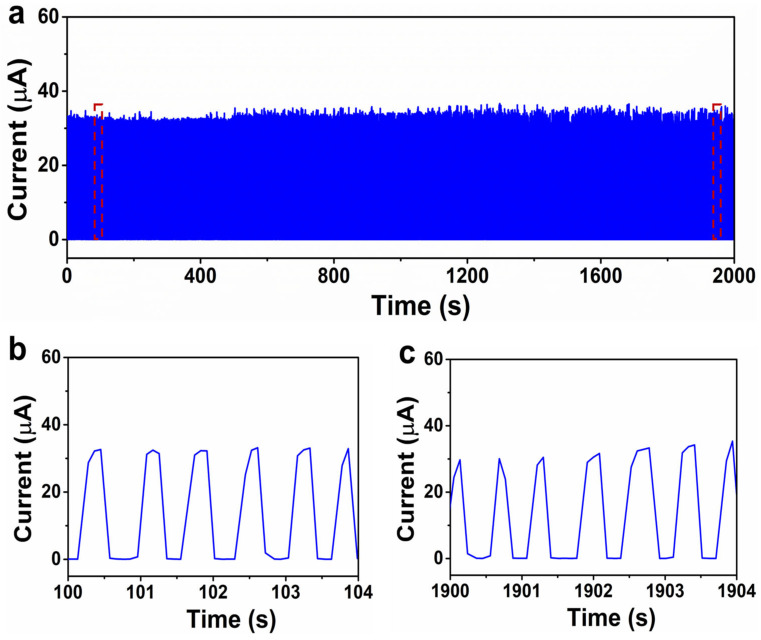
(**a**) Durability of the tactile sensor to dynamic pressure (10 kPa) up to 3000 cycles. (**b**) and (**c**) Enlarged view of the curves shown in (**a**).

To study the current response of the tactile sensors to the external pressure in detail, the tactile sensors are characterized by a test system, including a digital source meter, test stand, and force gauge. Firstly, the current change of the tactile sensors to diverse pressures was characterized as shown in Figure 1f. It is shown that the tactile sensor based on the flat PEDOT:PSS film has poor sensitivity, which is attributed to the limited deformation of the polymer film under external pressure. It is observed that the tactile sensors based on the microstructured ZnO/PEDOT:PSS composite film show an excellent response to the external pressure, which means that the surface microstructures are beneficial to obtain the high sensitivity. For practical applications, a fast response and relaxation time is highly desired. Here, the response speed was also characterized as shown in Figure 1g. It is shown that the tactile sensor has a response time and relaxation time of 25 ms and 35 ms, respectively, which is comparable with the results reported by other research groups [1,10]. The dynamic pressure response of the tactile sensors is important to robotics for texture recognition. As shown in Figure 1h, the tactile sensors can distinguish the altitudes of different dynamic pressures and repeatable peaks for the same dynamic pressure. Additionally, regarding the durability of the tactile sensors also characterized as shown in Figure 2a–c, it can be observed that the tactile sensor shows negligible current fluctuation (<10%) up to 3000 loading/unloading cycles, showing potential for practical applications. The sensing performance of our tactile sensors with that of the previously reported works is listed in Table 1.

### 3.3. The Tactile Sensor for Real-Time Health Monitoring

**Figure 3:**
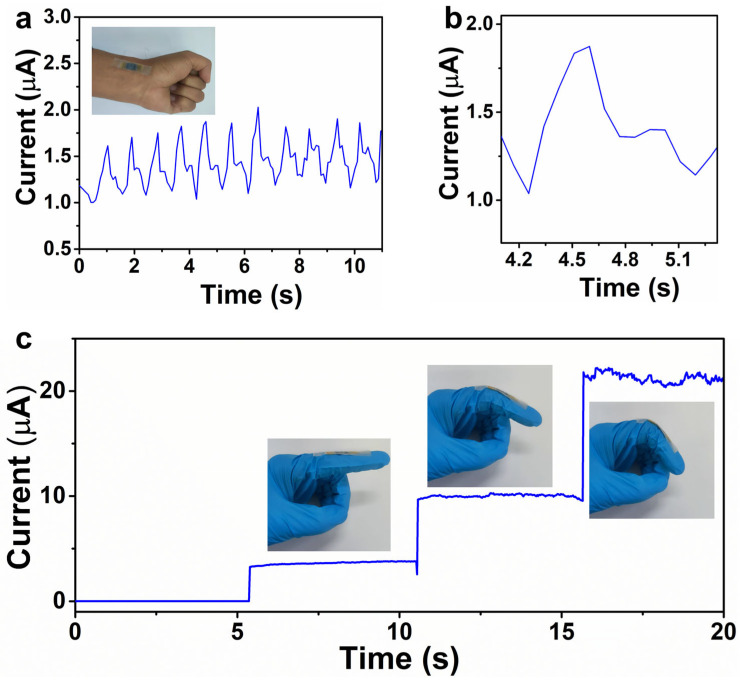
(**a**) Real-time monitoring of wrist pulses. Inset is the photograph of a tactile sensor attached to the volunteer’s wrist. (**b**) Enlarged view of the pulse waveform picked from (**a**). (**c**) Current response of the tactile sensor when the finger is under small, medium, and large bending angles, respectively.

Cardiovascular disease is one of the most serious diseases, which can cause a series of health problems. Early recovery from cardiovascular problems is relevant to providing useful information for treatment. The wrist pulse waveform can reveal the blood vessel wall thickening and arterial stiffness change by three typical peaks: tidal waves (T-wave), diastolic wave (D-wave), and percussion waves (P-wave). To demonstrate the potential of our tactile sensor in monitoring the wrist pulses in real time, the device is attached to the radial artery of a volunteer’s wrist with the assistance of a medical pressure tape. As shown in Figure 3a,b, the tactile sensor can detect the wrist pulses in detail. The heart beat rate per minute (bpm) of a 33-year-old male is 70, which belongs to the normal bpm range of a healthy adult (60–100). Compared with the previous works, the wrist pulse signals can be more clearly revealed by the mold-free ZnO/PEDOT:PSS tactile sensors [31,32,33]. By integrating our flexible tactile sensors into wearable electronics, it will be an excellent candidate for real-time health monitoring in the future.

Additionally, our tactile sensor is applied to detect the finger-bending states of a volunteer, demonstrating its potential for future smart sport. As shown in Figure 3c, the tactile sensor is attached to the outer side of the index finger to monitor its bending states. When the finger is in a flat state, there is a negligible current for the tactile sensor, and the current flowing through the device increases with the bending angles as shown in Figure 3c. When the index finger is in the flat state, there is almost no external pressure applied to the device, thereby creating significant resistance for the tactile sensor. The contact area between the composite films will increase when the bending angle increases, resulting in an enlarged current. The results demonstrate that the tactile sensor has great potential in detecting the gestures of the human body.

### 3.4. Tactile Sensor Array for Spatial Pressure Mapping

**Figure 4:**
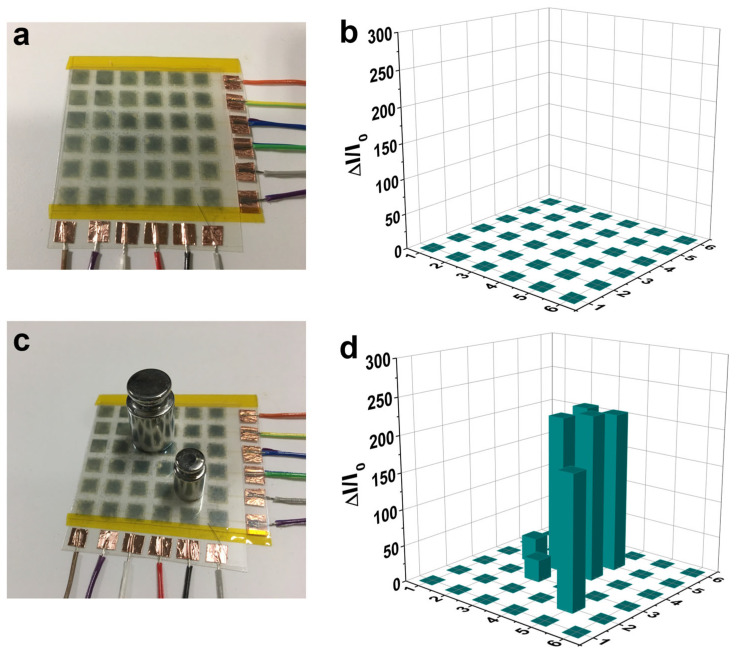
(**a**) Photo image of a 6 × 6 tactile sensor array. (**b**) Current response of the tactile sensor arrays when there is no pressure applied. (**c**) Photo image of the tactile sensor arrays with two weights (20 g, 5 g) loaded on top. (**d**) Current responses of the tactile sensor arrays to the loaded weights.

Tactile sensor arrays with high sensitivity and excellent flexibility are highly required as e-skin for robots and human–machine interfaces. As shown in Figure 4, all the tactile pixels show negligible current without external pressure, indicating the tactile sensor has low-power consumption at this state. When different weights are put on top of the tactile sensor arrays, the spatial pressure distribution is well revealed, demonstrating its promising potential for e-skin application. Furthermore, the tactile sensor arrays are semitransparent, indicating their potential applications for touchable displays.

## 4. Conclusions

In summary, a mold-free approach is presented to develop microstructured ZnO/PEDOT:PSS for highly sensitive tactile sensors. The sensors are demonstrated with a high sensitivity of 4 × 10^3^ kPa^−1^ in the pressure range from 0–10 kPa, a fast response time of 25 ms, and long-term durability up to 3000 cycles. Thanks to its excellent sensing performance, the tactile sensor can detect human wrist pulses in real time, which is attractive for wearable healthcare electronics. Additionally, the tactile sensors could detect subtle human body motions and could be integrated to tactile sensor arrays to detect spatial pressure, demonstrating their potential application as e-skin. High-density pressure sensor arrays are highly desired for e-skin applications. In the following work, the sensing area of every pixel is expected to be minimized by optimizing the laser etching parameters. This work may inspire the development of ultra-thin and high-performance tactile sensors.

## Figures and Tables

**Table 1:** Performance of reported flexible tactile sensors.

Materials	Sensitivity	Response Time	Durability [Cycles]	Ref.
polyurethane sponge/graphene	1.7356 kPa^−1^ at 0–55 kPa	147 ms	2000	[22]
CNTs	1150.9 kPa^−1^ at 0–50 Pa	43 ms	>2000	[23]
MXene	175 kPa^−1^ at 0–10 kPa	0.43 s	5000	[24]
GNW	2297.47 kPa^−1^	9 ms	10,000	[25]
MWNTs/PDMS	1.619 kPa^−1^	80 ms	1000	[26]
(3-aminopropyl) triethoxysilane-enhanced CNPs/carboxylated MWCNTs/cellulosic fiber composites	1.0005 kPa^−1^	40 ms	500	[27]
AgNPs/CNTs	20 kPa^−1^ at 0–2 kPa	48 ms	6000	[28]
MXene/ZnO	236.5 kPa^−1^	100 ms	10,000	[29]
MXene/PDMS	519 kPa^−1^	62.7	500	[30]
ZnO/PEDOT:PSS	4 × 10^3^ kPa^−1^ within pressure range 0–10 N	25 ms	3000	this work

## Data Availability

The original contributions presented in this study are included in the article. Further inquiries can be directed to the corresponding author.
